# The Moderating Effect of Peer Attachment on Associations Between Children’s and Adolescents’ Cyberbullying Victimization, Bystanding, and Depressive Symptoms

**DOI:** 10.3390/ijerph22010008

**Published:** 2024-12-25

**Authors:** Michelle F. Wright

**Affiliations:** Department of Psychology, Indiana State University, Terre Haute, IN 47809, USA; michelle.wright@indstate.edu; Tel.: +1-812-237-2446

**Keywords:** cyberbullying, age, gender, depression, peer attachment

## Abstract

The primary objective of this short-term longitudinal study was to investigate how age groups affect the relationships between cyberbullying victimization, bystanding, and depression among a convenience sample of students across different educational levels; there was a total of 234 elementary school students (fourth and fifth graders), 363 middle school students (sixth to eighth graders), and 341 high school students (ninth to twelfth graders) from the United States who completed self-reported questionnaires on cyberbullying, depression, and peer attachment during 2020. Additionally, this study examined whether peer attachment acted as a moderator in these relationships. The results revealed that strong peer attachment significantly moderated the connections between cyberbullying involvement and depression, as measured six months later, with particularly pronounced effects among middle school students. In contrast, weaker peer attachment intensified the positive correlations between cyberbullying victimization, bystanding, and subsequent depression. These findings highlight the crucial role of cultivating strong peer relationships, especially during pivotal developmental phases such as middle school. Implementing programs that promote positive peer interactions and supportive networks can be effective at alleviating the psychological effects of cyberbullying.

## 1. Introduction

In today’s world, information and communication technologies (ICTs) play a vital role in everyday life, offering both advantages and potential dangers [1,2]. Among the most notable risks of ICT use is cyberbullying, which has gained significant attention due to its harmful effects on mental health [3,4,5,6]. As the link between cyberbullying—whether as a victim, perpetrator, or bystander—and depression is well documented, researchers have begun exploring elements that could reduce these negative impacts. Factors like age group and peer attachment are seen as crucial in shaping the influence of cyberbullying on mental health. However, despite their relevance, studies focusing on age-related differences in cyberbullying experiences and the role of age in this context remain sparse. Additionally, while depression is commonly linked to cyberbullying across all age groups, it is unclear whether this link differs by age [3,4,5,6]. Consequently, this research aims to explore the differences in cyberbullying experiences across various age groups. Furthermore, it examines how age group variations affect the moderating role of parental mediation in the relationship between cyberbullying and depression, as assessed six months later. This research adds to the current literature on peer attachment and its importance at various developmental stages, as well as how it alleviates negative experiences associated with cyberbullying.

### 1.1. Cyberbullying and Age

Cyberbullying, which includes witnessing, experiencing, or participating in repeated aggressive behaviors through ICTs, resembles traditional face-to-face bullying in many ways [5,7,8,9]. Typically, there is a power imbalance between the bully and the victim, amplified by technology. These behaviors can manifest in various forms, such as spreading rumors, harassment, social exclusion, humiliation, physical threats, gossip, verbal abuse, hacking, sending anonymous or explicit content, identity threats, and harassment through instant messaging, social media, or text [10,11].

Given the age-related differences observed in face-to-face bullying, age has been proposed as a potential predictor of cyberbullying behavior. Younger children in elementary school (ages 9 through 11) tend to display more physical aggression, while middle school (ages 11 through 14) students engage more in verbal and relational aggression as their social skills and peer interactions develop [12]. As an indirect form of bullying, cyberbullying may similarly peak during adolescence and decline in late adolescence and young adulthood [13]. However, this trend is complicated by studies that associate higher ICT use with increased exposure to cyberbullying among children and adolescents [3,10,14,15]. Few studies have longitudinally examined how age affects the perpetration, victimization, or witnessing of cyberbullying over time [16].

Research shows that early adolescents are most vulnerable to cyberbullying victimization, with rates that are higher than in younger children, late adolescents, or young adults [16,17]. For example, hacking-related cyberbullying tends to peak during middle school and decrease in high school [18]. Despite these findings, age alone has proven to be an inconsistent predictor of cyberbullying involvement. Wade and Beran’s research revealed variations, with cyberbullying perpetration and victimization peaking among ninth graders while showing a decline in younger middle school students [19]. The relationship between age and cyberbullying bystander rates remains underexplored, although it is possible that patterns similar to those in cyberbullying victimization may apply. Younger adolescents, especially those in middle school, may be more likely to witness cyberbullying due to their stage of development and the significance of peer relationships at that age [14,15,16,17,18]. As middle school students are at a critical stage of social development, they may be more prone to engaging in bystander behavior, whether passive or active, compared with older adolescents who possess more developed coping skills and a stronger sense of identity.

Factors such as the amount of time spent online and the types of technologies used may explain cyberbullying patterns more effectively than age alone, as many studies do not account for these variables. Additionally, the interaction between gender and age is often overlooked in cyberbullying research, limiting a comprehensive understanding of its developmental dynamics. In conclusion, while age plays a role in cyberbullying trends, it is a complex factor that interacts with technological use and social development. This complexity underscores the need for more nuanced research to better understand the relationships between age, technology, and cyberbullying behaviors.

### 1.2. Depression

Cyberbullying victimization and witnessing (bystanding) have been linked to increased rates of depression [13]. The negative effects of cyberbullying extend beyond mental health, impacting academic performance and behavior, with both victims and bystanders reporting lower academic achievement and more internalizing issues, such as anxiety and withdrawal [3,4,5,6]. Adolescents who are frequently cyberbullied may begin to feel powerless in stopping the harassment, leading to learned helplessness, a theory that explains how repeated exposure to such events can foster feelings of futility and depression in both victims and bystanders [20]. This helplessness can contribute to a decline in self-esteem, as the individual starts to believe that their efforts to stop the bullying are ineffective. Similarly, bystanders who witness cyberbullying but feel unable to intervene may also experience learned helplessness, eventually becoming passive and reluctant to assist or report the bullying.

Many studies have overlooked the role of traditional, face-to-face bullying, a related factor that can further complicate the link between cyberbullying and depression [13,14,15,21]. Victims of cyberbullying frequently display more internalizing issues, such as depression and anxiety, and engage in externalizing behaviors, like substance abuse and aggression [4,5,6,7]. Moreover, research indicates a positive association between cyberbullying involvement and academic challenges, such as poor performance, absenteeism, and disciplinary problems at school [18]. Earlier critiques of cyberbullying research highlighted the failure to account for face-to-face bullying, which could skew the reported associations between cyberbullying and negative outcomes [17]. Recent studies have sought to address this gap by examining the combined effects of both cyberbullying and traditional bullying. Findings show that individuals who experience both types of bullying suffer from more severe internalizing issues compared with those who experience just one form of victimization [18]. Additionally, contemporary research now considers face-to-face bullying when analyzing the psychological, academic, and behavioral impacts of cyberbullying. For example, Bonanno et al. [21] demonstrated that cyberbullying, both as a perpetrator and as a victim, was independently linked to higher rates of depression and suicidal ideation. Therefore, recognizing the overlap and unique consequences of both cyberbullying and traditional bullying is essential for understanding their comprehensive effects on the mental health and well-being of children and adolescents.

### 1.3. Peer Attachment

Peer attachment refers to adolescents’ internalized beliefs about the availability and responsiveness of their peers [22]. When adolescents encounter difficulties in their peer relationships, they are more likely to experience conflicts, increasing the likelihood of peer victimization and behavioral issues [23,24]. Studies confirm that poor peer relationships are linked to higher rates of victimization and problem behaviors [25,26]. Adolescents who struggle to socially integrate within their peer groups are at greater risk of rejection and victimization. Additionally, difficulties in peer relationships can reduce empathy, contributing to behavioral problems and aggressive tendencies [27]. On the other hand, strong peer attachment and social integration foster empathy and sympathy, which are associated with lower levels of delinquency and aggression [28]. High levels of peer attachment have also been negatively correlated with both face-to-face bullying and cyberbullying perpetration and victimization.

Research shows that peer attachment evolves developmentally as children grow. Initially, young children form attachments with caregivers, but as they reach school age and adolescence, peers become increasingly significant [29,30]. During middle childhood (ages 6–12), children begin to form closer friendships and increasingly depend on peers for emotional support and social activities. Peer attachment at this stage is often characterized by loyalty, trust, and shared experiences [31]. As children enter adolescence (ages 12–18), peer attachment becomes more nuanced, with teens seeking both autonomy and close peer connections. During this period, attachment figures often shift from parents to peers, and friendships play a crucial role in identity development and emotional well-being [32]. Although much of the research on peer attachment focuses on adolescence, there is a growing body of work examining attachment across various developmental stages. This literature sheds light on how peer relationships change over time and sheds light on their influence on emotional development and well-being. However, despite these insights, limited research exists on whether age impacts how peer attachment moderates the relationships among cyberbullying victimization, bystander behavior, and depression, leaving gaps in our understanding of these dynamics across developmental stages.

### 1.4. Theoretical Framework

Bronfenbrenner’s ecological systems theory offers a thorough framework for understanding the various environmental systems that shape adolescent development. According to this theory, development is influenced by interactions within and between multiple environmental layers, ranging from the immediate microsystem (e.g., family, peers) to the larger macrosystem (e.g., societal norms, cultural values) [33]. In this study, the microsystem includes environments that directly impact the students, such as peer groups and school settings, which shape their experiences with cyberbullying and peer attachment. The mesosystem refers to the relationships between elements of the microsystem, such as the link between school dynamics and family support, which may affect how adolescents respond to cyberbullying. The exosystem involves external factors that indirectly influence the students, such as school policies on cyberbullying and social media regulations. The macrosystem encompasses the broader cultural and societal views on cyberbullying and mental health. Finally, the chronosystem accounts for the dimension of time, including changes and transitions in students’ lives, such as progressing from middle to high school. Each of these systems may play a role in how age group and peer attachment shape the relationship between involvement in cyberbullying and depression.

Additionally, the theory of learned helplessness may provide insights into the connection between cyberbullying victimization, bystanding, and depression [20]. Incorporating learned helplessness into the study allows researchers to explore the psychological processes driving the relationship between cyberbullying experiences and depression. This perspective emphasizes the importance of interventions that empower victims and bystanders, strengthen peer support, and break the cycle of helplessness.

The primary goal of this study was to investigate the connection between cyberbullying and depression. The second aim was to determine how perceived peer attachment moderates this relationship, with depression being measured again six months later (Time 2). Based on prior research on cyberbullying, depression, and age-related changes in peer attachment, it was hypothesized that higher peer attachment may reduce the positive relationship between cyberbullying involvement and depression (Hypothesis 1). Conversely, lower peer attachment could intensify these relationships (Hypothesis 2).

## 2. Materials and Methods

### 2.1. Participants

This study utilized a random stratified sample of 935 participants living in middle-class suburbs in a Midwestern U.S. city. The sample was composed of 234 elementary school students, with an average age of 10.43 years (SD = 1.10), drawn from 4th grade (*n* = 100) and 5th grade (*n* = 134). Additionally, 363 middle school students participated, with a mean age of 13.03 years (SD = 0.13), including 6th grade (*n* = 105), 7th grade (*n* = 115), and 8th grade (*n* = 143). The high school cohort included 341 students, with an average age of 16.69 years (SD = 0.67), representing 9th grade (*n* = 86), 10th grade (*n* = 76), 11th grade (*n* = 82), and 12th grade (*n* = 95). In terms of racial demographics, the majority of participants identified as white (60%), followed by Latinx (30%), Black/African American (5%), Asian (1%), and other (4%). Income data were not collected.

A small to medium effect size (Cohen’s d = 0.30) was anticipated in the relationships between cyberbullying victimization, bystanding, and depression, moderated by peer attachment. To reduce the chance of Type I errors, a significance level of 0.05 was selected. Additionally, a power level of 0.80 was chosen, ensuring an 80% probability of correctly rejecting the null hypothesis if it is false, thereby reducing the likelihood of Type II errors. Based on these parameters, a power analysis suggested a minimum sample size of approximately 200 participants would be necessary to detect the expected effect sizes with adequate power. Given the study’s inclusion of three distinct age groups—elementary (4th and 5th grades), middle (6th to 8th grades), and high school (9th to 12th grades)—a target of at least 200 participants per group was recommended. This would result in a total minimum sample size of 600 students to ensure sufficient statistical power to detect significant main effects and interaction effects. Therefore, with 935 participants overall and at least 200 in each age group, the sample size was deemed sufficient for robust analysis.

### 2.2. Procedures

Prior to data collection, ethical approval was secured in accordance with APA guidelines. From an initial pool of 75 school districts, five were randomly chosen using a Microsoft Excel randomization function. Out of these, three districts were unavailable due to prior commitments, one failed to respond, and one district expressed interest and granted permission for the study. Within this approved district, one elementary school, one middle school, and one high school were selected to participate. No incentives were offered to students for their participation in the study. Meetings were held with the school principals, followed by classroom announcements that outlined the study’s purpose and expectations. Parental consent forms were distributed to elementary and middle school students, while informed consent documents were provided to high school students aged 18 and older. Specifically, 306 permission slips were given to elementary students, 460 to middle school students, and 400 to high school students, in addition to 30 informed consent forms for those 18 and older.

For elementary school students, 60 permission slips were not returned, 12 were returned without permission, and 234 were returned with permission. In the middle school group, 67 slips were not returned, 20 denied permission, and 373 slips granted permission. In the high school group, 49 slips were not returned, 10 were returned without permission, 330 granted permission, and 11 informed consents were returned. Data collection occurred in fall 2019. On the scheduled day, 10 middle school students were absent, resulting in 363 participants from that group. Students completed assent forms and paper-and-pencil questionnaires during regular school hours, providing demographic information (e.g., age, gender, technology use) and responding to questions related to face-to-face bullying involvement, cyberbullying involvement, depression, and parental mediation of technology use. All present students gave their consent to participate in the study.

In spring 2020, a reminder letter was sent to the parents or guardians of the participants, informing them of their child’s involvement in the study six months earlier and the importance of participating again. If parents or guardians did not wish for their child to participate a second time, they were instructed to write their child’s first and last name on the letter and return it to the school. No letters were returned. On the second day of data collection, students were once again provided with assent forms and a paper-and-pencil questionnaire focused on depression. All students agreed to participate for a second round. However, three 12th-grade students were absent on the data collection day due to prior commitments and were also unavailable for makeup sessions, resulting in their exclusion from the final dataset.

### 2.3. Measures

#### 2.3.1. Face-to-Face Bullying and Cyberbullying Victimization and Bystanding

Participants’ engagement with technology was measured using a set of ten items (e.g., frequency of texting), each rated on a scale from 1 (never) to 5 (all the time) [34]. The responses were aggregated into a composite score, with higher values reflecting more frequent technology use. The internal consistency of this measure, indicated by Cronbach’s alpha, ranged between 0.80 and 0.91.

#### 2.3.2. Depression

Participants’ depressive symptoms over the past two weeks were assessed using the 20-item Center for Epidemiological Studies Depression Scale [35], which evaluates emotions such as sadness and changes in appetite (e.g., “I did not feel like eating, my appetite was poor”). Responses were rated on a scale from 0 (rarely) to 3 (most or all of the time). This questionnaire was administered at two different time points. The scale demonstrated strong internal consistency, with Cronbach’s alphas ranging from 0.80 to 0.88 at both Time 1 and Time 2.

#### 2.3.3. Peer Attachment

The Inventory of Parent and Peer Attachment questionnaire [36] evaluated how adolescents perceive their relationships with peers (e.g., “My friends understand me”). Participants rated 25 statements on a scale of 1 (almost never or never true) to 5 (almost always or always true). The reliability, assessed through Cronbach’s alpha, ranged between 0.84 and 0.89.

### 2.4. Analytic Plan

A single multigroup comparison structural equation model was utilized for the analysis. This model incorporated the robust maximum likelihood estimator and the full information maximum likelihood method to address missing data, which comprised about 0.5% of the dataset (twenty-two incomplete records: fifteen from elementary school, five from middle school, and two from high school), using Mplus Version 8.10. Additional pathways were established from cyberbullying victimization and bystander roles to peer attachment and depression at Time 2. Although gender was initially considered a predictor, it was found to be non-significant and was thus excluded from further analyses. Two-way interactions between peer relationships and experiences of cyberbullying were explored through simple slopes analysis. Additionally, technology use was examined as a predictor of cyberbullying victimization and bystander roles while controlling for face-to-face bullying victimization and bystander roles across all types of cyberbullying involvement. To account for prior levels of depression, depression at Time 1 was included as a predictor for depression at Time 2.

## 3. Results

### 3.1. Correlations

Table 1 presents the Pearson correlations. The findings showed that peer attachment had a negative correlation with cyberbullying victimization, bystanding, and depression across all age groups. Conversely, cyberbullying victimization was positively correlated with both cyberbullying bystanding and depression. Additionally, there was a positive association between cyberbullying bystanding and depression.

### 3.2. Associations Among Cyberbullying Involvement, Peer Attachment, and Depression

The multigroup comparison structural model exhibited a good fit, with statistics indicating [χ^2^(816) = 799.30, *p* = 0.65, CFI = 0.99, TLI = 0.99, RMSEA = 0.04, SRMR = 0.04; see Table 2]. Across all age groups, peer attachment demonstrated negative correlations with cyberbullying victimization, bystanding, and depression at Time 2. Additionally, both cyberbullying victimization and bystander roles were positively associated with Time 2 depression, with notably stronger correlations being observed among middle school students compared with those in high school and elementary school. Depression at Time 1 was found to be a positive predictor of depression at Time 2 across all age groups.

Interaction effects were analyzed between cyberbullying victimization and peer attachment, as well as between cyberbullying bystanding and peer attachment. Further investigation revealed that higher levels of peer attachment reduced the positive associations between cyberbullying victimization, bystanding, and Time 2 depression, while lower levels of peer attachment amplified these relationships. These patterns were consistent across all age groups, with middle school students displaying stronger associations.

## 4. Discussion

This study explored the relationships between cyberbullying victimization, bystander roles, peer attachment, and depression, measured six months later across various age groups (elementary, middle, and high school). It specifically investigated how peer attachment influences the connections between experiences of cyberbullying and Time 2 depression while controlling for Time 1 depression. The research emphasizes the potential protective effect of robust peer relationships. Given the harmful impact of cyberbullying on the mental health of children and adolescents, the findings highlight the necessity of parental intervention strategies and school-based programs that foster supportive peer environments.

This study confirmed positive associations between cyberbullying victimization, bystander roles, and Time 2 depression, consistent with previous research findings [37,38,39,40]. It further extends these findings by emphasizing how bystander experiences can exacerbate psychological distress. The theory of learned helplessness [20] provides a framework for understanding these associations, indicating that feelings of powerlessness and lack of control during cyberbullying incidents significantly contribute to the onset and persistence of depression in adolescents.

Peer attachment showed a consistent negative relationship with cyberbullying victimization and bystanding across all age groups, even when controlling face-to-face bullying involvement. This suggests that stronger beliefs in the availability and responsiveness of peers can reduce the likelihood of both experiencing and witnessing cyberbullying. These findings highlight the importance of positive peer relationships in mitigating conflicts, including cyberbullying, thereby enhancing children’s and adolescents’ ability to navigate peer interactions successfully and reduce interpersonal discord [41]. High levels of peer attachment correlate with increased empathy towards peers [42], and the existing literature indicates that empathy diminishes the likelihood of involvement in cyberbullying among children and adolescents [43].

Across all age groups, peer attachment served as a buffer against the depression associated with being victimized or witnessing cyberbullying, with the most significant impact being observed among middle school students, supporting Hypotheses 1 and 2. Peer relationships are particularly valued by many U.S. adolescents, who prioritize maintaining these connections [44]. Disruption of such relationships could lead to increased peer conflict, which is generally unfavorable for adolescents as it can affect their social networks and standing [45]. Our findings suggest that peer attachment plays a crucial protective role against both victimization and bystanding in cyberbullying, underscoring the essential function of positive peer interactions in reducing adolescents’ vulnerability to cyberbullying behaviors. While much of the peer attachment research focuses on adolescence, this study broadens the existing literature by examining attachment across different developmental stages, providing valuable insights into the evolution of peer relationships and their role in alleviating negative experiences like cyberbullying.

The relationships identified were consistent across all age groups, with particularly strong associations being found among middle school students. Previous research shows that early adolescents experience and perpetrate cyberbullying at higher rates [16], and our study highlighted the pronounced effects within this age group. However, other studies [17] have reported varying involvement levels across different age groups, likely due to methodological differences in measuring cyberbullying. Notably, our study improved rigor by controlling factors such as technology use and face-to-face bullying, enhancing the robustness of previous research methodologies.

The study’s findings can be interpreted through Bronfenbrenner’s ecological systems theory, which emphasizes the significance of various environmental systems in shaping adolescent development [33]. The strong peer attachments that moderated the relationship between cyberbullying involvement and depression align with the microsystem’s focus on immediate social contexts, such as peer groups and school settings. The pronounced impact of cyberbullying and the protective effect of peer attachment during middle school highlight the mesosystem, where interactions between home and school environments shape adolescents’ experiences. The varying influence of peer attachment across different age groups reflects the exosystem’s role in indirectly influencing these dynamics through policies and societal norms. Finally, considering developmental stages, such as middle school, relates to the chronosystem, which underscores the importance of time and transitions in understanding adolescents’ vulnerability to cyberbullying. Overall, this study reinforces the idea that fostering strong, supportive peer relationships within these environmental contexts can mitigate the adverse psychological effects of cyberbullying, especially during critical developmental periods [44,45].

### 4.1. Practical Solutions

Schools can play a vital role in addressing cyberbullying by fostering positive peer relationships and promoting mental health. For younger students, peer mentorship programs and empathy-building activities, like storytelling and role-playing, can create a supportive environment. Middle schools can implement peer support networks, social media literacy programs, and counselor-led groups to teach responsible online behavior and provide emotional support. High schools can empower students through peer-led workshops, monitored online platforms, and mental health integration in the curriculum, emphasizing the impact of peer attachment on well-being. Across all age groups, schools should adopt comprehensive anti-cyberbullying policies, celebrate positive peer interactions through events like “Friendship Week,” and provide access to anonymous counseling services. By focusing on peer attachment and proactive intervention, schools can help reduce the negative effects of cyberbullying on students’ mental health.

### 4.2. Study Limitations and Future Research

One limitation of this study is the reliance on self-reported data, which may introduce biases such as social desirability or inaccurate recall, particularly when assessing sensitive topics like cyberbullying and peer attachment. Additionally, this study focuses on correlational relationships and utilizes a short-term longitudinal design, which limits the ability to establish causation between cyberbullying victimization, bystanding, peer attachment, and subsequent depression. Furthermore, the findings may not generalize across diverse cultural or socioeconomic contexts as the study’s sample may not reflect the variability in access to technology, peer support, and school resources.

Future research should employ longitudinal designs over multiple years to better understand the causal pathways linking cyberbullying, peer attachment, and mental health outcomes across developmental stages. Expanding the sample to include diverse populations will allow for the exploration of cultural and contextual factors that influence these dynamics. It would also be beneficial to incorporate multi-informant approaches, gathering data from parents, teachers, and peers to provide a more comprehensive understanding of peer attachment and cyberbullying experiences. Finally, future studies could examine the effectiveness of specific school-based interventions aimed at strengthening peer attachment and reducing the impact of cyberbullying on mental health, tailoring strategies to the unique needs of different age groups.

## 5. Conclusions

This study delved into the intricate relationships among cyberbullying victimization, bystanding, peer attachment, and Time 2 depression while also investigating how peer attachment influences these dynamics across different age groups (elementary, middle, and high school). The findings underscore the critical role of positive peer relationships in mitigating the adverse impacts of cyberbullying on children and adolescents’ mental health. Given the protective effects of peer attachment, it is crucial to identify strategies that can effectively reduce the negative consequences of cyberbullying, particularly depression. Schools play a pivotal role in nurturing peer relationships and attachment, which are instrumental in minimizing the detrimental effects of cyberbullying victimization and bystanding on young people’s well-being, especially as these dynamics vary across age groups. This study contributes to the existing literature by highlighting the significance of peer attachment across developmental stages and its potential to alleviate negative experiences associated with cyberbullying.

## Figures and Tables

**Table 1:** Correlations among peer attachment, cyberbullying victimization, cyberbullying bystanding, and Time 1 and Time 2 depression.

	1	2	3	4	5
1. Peer Attachment	---				
2. CBV	−0.27 **	---			
	−0.36 ***				
	−0.30 ***				
3. CBB	−0.20 *	0.26 **	---		
	−0.29 ***	0.31 ***			
	−0.24 *	0.28 **			
4. T1 Depression	−0.31 ***	0.31 ***	0.28 **	---	
	−0.38 ***	0.36 ***	0.34 ***		
	−0.30 ***	0.30 ***	0.28 **		
5. T2 Depression	−0.33 ***	0.31 ***	0.29 ***	0.43 ***	---
	−0.38 ***	0.37 ***	0.35 ***	0.51 ***	
	−0.31 ***	0.31 ***	0.28 **	0.42 ***	

Notes. CBV stands for cyberbullying victimization, CBW refers to cyberbullying bystanding, T1 indicates Time 1, and T2 represents Time 2. The first number relates to elementary school students, the second to middle school students, and the third to high school students. * *p* < 0.05, ** *p* < 0.01, *** *p* < 0.001.

**Table 2:** Multigroup comparison of the associations among peer attachment, cyberbullying victimization, cyberbullying bystanding, and Time 2 depression by age group.

Age Group	Predictors	Time 2 Depression	
		β	*SE*
Elementary School	Peer Attachment (PA)	−0.25 **	0.10
	CBV	0.20 *	0.11
	CBB	0.18 *	0.08
	PA × CBV	−0.10 *	0.04
	PA × CBB	−0.09 *	0.01
Middle School	Peer Attachment (PA)	−0.32 ***	0.13
	CBV	0.31 **	0.12
	CBB	0.30 **	0.12
	PA × CBV	−0.11 *	0.13
	PA × CBB	−0.11 *	0.11
High School	Peer Attachment (PA)	−0.27 **	0.10
	CBV	0.26 **	0.09
	CBB	0.23 *	0.10
	PA × CBV	−0.08 *	0.12
	PA × CBB	−0.08 *	0.08

Notes. CBV refers to cyberbullying victimization, while CBB stands for cyberbullying bystanding. The first number pertains to elementary school students, the second to middle school students, and the third to high school students. Time 1 depression was a predictor of Time 2 depression for elementary school (β = 0.34, *p* < 0.001), middle school (β = 0.39, *p* < 0.001), and high school participants (β = 0.35, *p* < 0.001). * *p* < 0.05, ** *p* < 0.01, *** *p* < 0.001.

## Data Availability

Data can be requested from the first author.
